# Xanthogranulomatous Pyelonephritis Presenting as a Psoas Abscess

**DOI:** 10.7759/cureus.21967

**Published:** 2022-02-07

**Authors:** Carlota Mendonça, Sérgio Freitas, Sara Jesus

**Affiliations:** 1 Family Medicine, Hospital Central do Funchal - Serviço de Saúde da Região Autónoma da Madeira, Entidade Pública Empresarial da Região Autónoma da Madeira (SESARAM, EPERAM), Funchal, PRT

**Keywords:** lumbago, abcess, psoas, pyelonephritis, xanthogranulomatous

## Abstract

Xanthogranulomatous pyelonephritis (XGP) is a rare form of severe pyelonephritis. It is characterized by progressive parenchymal destruction caused by chronic urinary tract obstruction and infection, typically resulting in a non-functioning enlarged kidney. Its presentation with a psoas abscess is infrequent, and only a few cases are described in the literature. Unlike the typical presenting symptoms of XGP, our patient presented classic symptoms of lumbago. Once the diagnosis was established, antibiotics were given and a nephrectomy was performed. Unfortunately, after the surgery, the patient developed mild monoparesis on the right lower limb with decreased knee extension muscle strength and needed a walking stick for support. Nonetheless, a delayed diagnosis could have been fatal.

## Introduction

Xanthogranulomatous pyelonephritis (XGP) was first described by Schlagenhaufer in 1916 and remains a rare disease with an annual incidence of 1.4 per 100,000 inhabitants [1-3]. XGP is an unusual variant of chronic pyelonephritis, characterized by progressive parenchymal destruction. Although the pathogenesis is unknown, urinary tract infection secondary to nephrolithiasis and obstruction must be present for XGP to develop [1], resulting in a non-functioning enlarged kidney [1,4].

History of recurrent urinary tract infection, diabetes, or kidney stones is a risk factor for the occurrence of XGP. It is more common in women than in men [4,5] with a wide range of ages, from newborns to the elderly [3-5], and the mean age of occurrence varies from 45 to 55.2 years [4]. The typical clinical presentation is flank or abdominal pain, lower urinary tract symptoms, fever, palpable mass, gross hematuria, and weight loss [4-6]. 

Para-renal tissue involvement is uncommon although some cases of psoas abscess have been reported [2,3,7,8]. Abdominal computed tomography scanning is the gold standard diagnostic method [1,2,4]. Most cases require a total nephrectomy to resolve symptoms [1,4,5].

## Case presentation

A 31-year-old Caucasian woman presented to the urgent clinic for lower back pain radiating to the right leg. Medical history was significant for depression and nonfunctioning right kidney secondary to large coraliform calculus, planned for nephrectomy. On observation, she had pain suggestive of lumbago and was prescribed analgesia. 

Seven days later, she attended an unscheduled appointment at the health care family unit. She maintained her complaints of lower back pain with irradiation to the right leg despite the prescribed analgesic medication. On observation, she was pale, afebrile (37,6ºC), anorexic, eupneic with normal oxygen levels (98%), normal blood pressure (123/81 mmHg) with tachycardia (115 beats per minute). Her cardiopulmonary exam was unremarkable. However, a 10 cm mass of firm consistency was palpable in the right flank, and the right iliac fossa had a painful-right positive lasegue sign. She was then referred to the hospital to the emergency room.

At the hospital, the laboratory showed normocytic normochromic anemia (hemoglobin {Hb} 7.6 g/dL), leucocytosis (white blood count 20800/mL), and increased C-reactive protein levels (278 mg/dL). Urinalysis was normal and urine and blood cultures were negative. Abdominal and pelvic computed tomography scans revealed aspects suggestive of xanthogranulomatous pyelonephritis and a posterior renal collection related to psoas abscess, probably of renal cause. She was admitted to the urology department.

**Figure 1:**
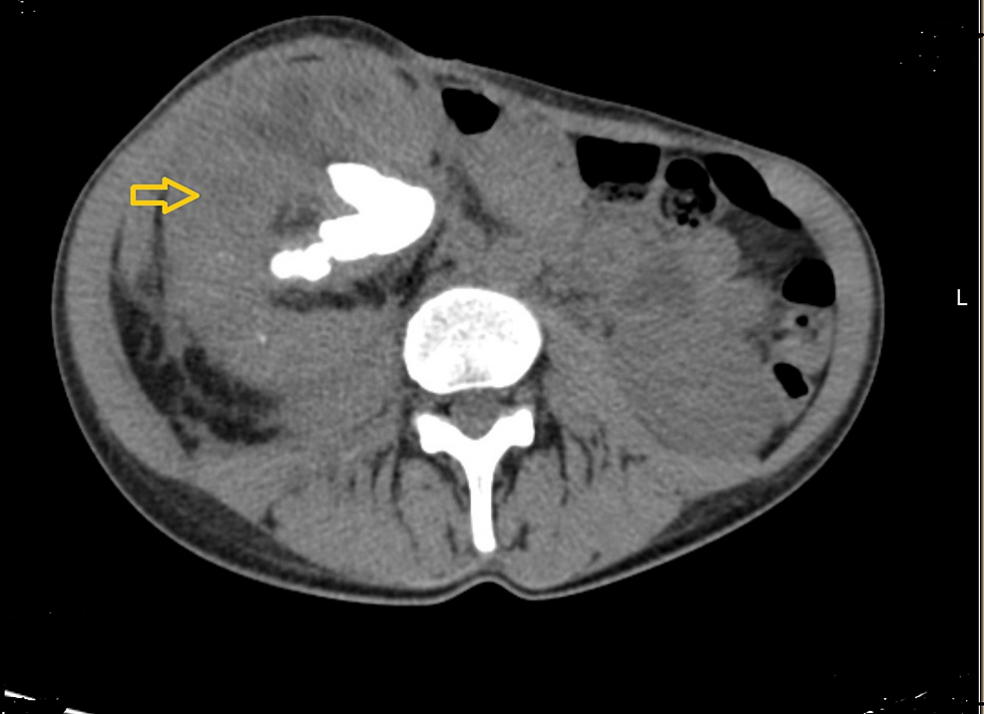
Abdominal and pelvic CT scan demonstrating kidney enlargement with calyceal dilatation

**Figure 2:**
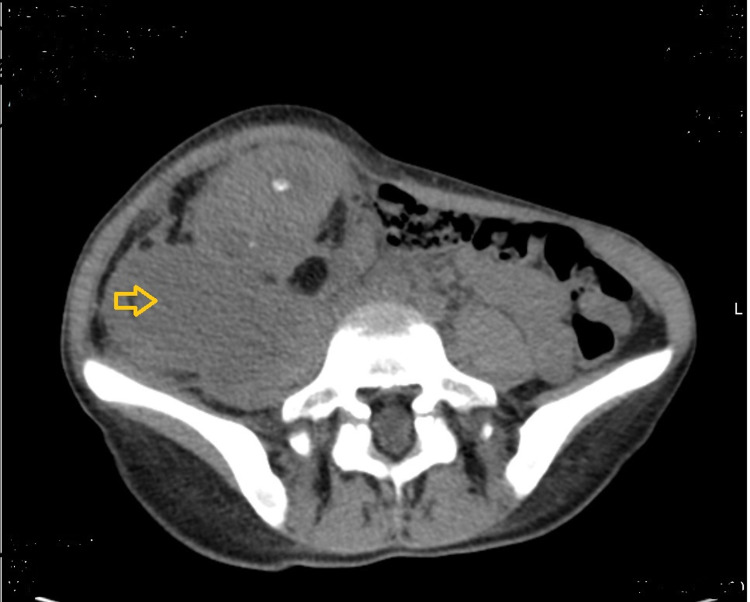
Abdominal and pelvic CT scan demonstrating psoas abscess collection

Percutaneous abscess drainage was performed and one unit of red blood cells was transfused. The chosen empiric antibiotic treatment regiment was intravenous cefuroxime and metronidazole. The bacteriological exam revealed *Proteus mirabilis* sensitivity to the prescribed antibiotic. The hospitalization was uneventful, with progressive improvement in inflammatory parameters and general health status. She was placed on the nephrectomy waiting list and was discharged on the 14th day.

Six months later she was admitted for a right-sided nephrectomy with a favorable clinical evolution, and eight days after surgery was discharged with oral antibiotics. After the surgery, the patient developed mild monoparesis on the right lower limb with decreased muscle strength in knee extension (a sequel of the right-sided radical nephrectomy). The electromyography revealed a chronic neurogenic lesion of the right quadriceps - femoral mononeuropathy and sensitive polyneuropathy axonal predominantly in the lower limbs with unresponsive external saphenous veins: decreased amplitude and sensitive conduction velocity of the median and cubital. She is undergoing physiotherapy and uses a walking stick for support.

## Discussion

Our patient was young, and even though she only complained of lower back pain with irradiation to the right leg, on observation, she presented other typical signs of XGP: she was pale, afebrile, had anorexia, and a painful right flank/iliac fossa on abdominal palpation. Usually, patients present with anemia and leukocytosis, as in our case [1,4,5]. The majority present with positive urine cultures, and only about 35% of patients have a sterile urine culture [1,4,5].

Psoas abscess is a relatively uncommon condition. Before discovering modern antituberculosis treatment, iliopsoas abscess was characteristically a well-recognized complication of tuberculosis of the spine [6]. However, with the decreasing prevalence of tuberculosis, iliopsoas abscess has become uncommon, and Crohn’s disease is the commonest cause of secondary iliopsoas abscess [3,6]. In addition, psoas abscess secondary to XGP is uncommon, and just a few cases are described in the literature [2,3,7,8]. The clinical presentation of iliopsoas abscess is often variable and non-specific, the classical clinical triad consisting of fever, back pain, and limp is present in only 30% of the patients [6]. Although our patient presented two of these classic symptoms of iliopsoas abscess, unlike the cases described in the literature of XGP with psoas abscess the majority had most of the typical presenting symptoms of XGP [2,3,7,8].

Abdominal and pelvic computed tomography scanning suggested the diagnostic of XGP with multiple rounded, low-density areas in ring-enhancing lesions in the renal parenchyma (the “bear paw” sign) and showed psoas abscess [1,4,5]. The definitive diagnosis of XGP is made by renal biopsy [1,7]. With or without adjunctive antibiotic therapy, nephrectomy has been the preferred treatment for XGP and is curative [1-5].

Unfortunately, after surgery, our patient developed mild monoparesis on the right lower limb with decreased knee extension muscle strength and needed a walking stick for support.

## Conclusions

In our patient's first assessment, it suggested lumbago. However, regarding the patient's medical history of right kidney large coliform calculus and the signs on observation (pale, subfebrile, anorexia, and a right flank/iliac fossa painful on abdominal palpation), a proper differential diagnosis was mandatory. This case reinforces the importance of a detailed approach of the patient instead of focusing merely on the symptoms. The delayed diagnosis and treatment likely contributed to the sequelae she now presents, mild monoparesis on the right lower limb requiring a walking stick for deambulation. However, the delayed diagnosis could have cost her life.

## References

[1] Dwivedi US, Goyal NK, Saxena V (2006). Xanthogranulomatous pyelonephritis: our experience with review of published reports. ANZ J Surg.

[2] Singh M, Ethiraj D, Indiran V, Kanase ND, Maduraimuthu P (2021). Xanthogranulomatous pyelonephritis with calculus migration into the psoas abscess: an unusual complication. Autops Case Rep.

[3] Alan C, Ataus S, Tunç B (2004). Xanthogranulamatous pyelonephritis with psoas abscess: 2 cases and review of the literature. Int Urol Nephrol.

[4] Li L, Parwani AV (2011). Xanthogranulomatous pyelonephritis. Arch Pathol Lab Med.

[5] Korkes F, Favoretto RL, Bróglio M, Silva CA, Castro MG, Perez MD (2008). Xanthogranulomatous pyelonephritis: clinical experience with 41 cases. Urology.

[6] Mallick IH, Thoufeeq MH, Rajendran TP (2004). Iliopsoas abscesses. Postgrad Med J.

[7] Kudalkar D, Remé P, Cunha BA (2004). Xanthogranulomatous pyelonephritis complicated by psoas abscess. Heart Lung.

[8] Kato K, Iwasaki Y, Kato Y, Kato K, Matsuda M (2018). Xanthogranulomatous pyelonephritis with psoas abscess and renocolic fistula. Clin Case Rep.

